# Supplementary material for: Single Cell Transcriptomic and Chromatin Profiles Suggest Layer Vb Is the Only Layer With Shared Excitatory Cell Types in the Medial and Lateral Entorhinal Cortex
Source: Front Neural Circuits. 2022 Jan 26;15:806154. doi: 10.3389/fncir.2021.806154 (PMC8826650; doi:10.3389/fncir.2021.806154)
Supplement: Supplementary file 14 [file Data_Sheet_1.ZIP › Inh_vs_Exc_Motifs/homerResults/motif16.similar.html]

motif16

## Information for motif16

C
A
G
T
T
A
C
G
G
C
T
A
G
C
T
A
C
G
A
T
C
T
A
G
T
A
C
G
T
C
G
A
T
C
G
A
C
G
T
A
C
G
A
T
A
C
G
T
  
Reverse Opposite:  

T
C
G
A
G
C
T
A
G
C
A
T
A
G
C
T
A
G
C
T
A
T
G
C
G
A
T
C
G
C
T
A
C
G
A
T
C
G
A
T
A
T
G
C
G
T
C
A
  

|  |  |
| --- | --- |
| p-value: | 1e-15 |
| log p-value: | -3.609e+01 |
| Information Content per bp: | 1.699 |
| Number of Target Sequences with motif | 834.0 |
| Percentage of Target Sequences with motif | 6.81% |
| Number of Background Sequences with motif | 1972.4 |
| Percentage of Background Sequences with motif | 5.11% |
| Average Position of motif in Targets | 320.7 +/- 247.3bp |
| Average Position of motif in Background | 339.4 +/- 281.5bp |
| Strand Bias (log2 ratio + to - strand density) | 0.1 |
| Multiplicity (# of sites on avg that occur together) | 1.05 |
| Motif File: | file (matrix) reverse opposite |

### Similar de novo motifs found

|  |  |  |  |  |  |  |  |
| --- | --- | --- | --- | --- | --- | --- | --- |
| Rank | Match Score | Redundant Motif | P-value | log P-value | % of Targets | % of Background | Motif file |
